# Development of a cellular model to study L-DOPA decarboxylase deregulation in the pathogenesis of schizophrenia

**DOI:** 10.1192/j.eurpsy.2023.1885

**Published:** 2023-07-19

**Authors:** A. Kurishev, D. Abashkin, D. Karpov, V. Golimbet

**Affiliations:** 1 Clinical Genetics Laboratory, Mental Health Research Center, Moscow, Russian Federation

## Abstract

**Introduction:**

Schizophrenia (SZ) is an inherited mental illness that affects 1% of the world’s population. Schizophrenia is a common functional psychosis with some unifying features that appears to have a universal distribution. Much of the research on SZ seeks to identify physiological, biochemical, or genetic features that differ between patients and healthy individuals. Biochemical factors represent an imbalance of certain biochemical substances in the brain, especially neurotransmitters. Early studies focused on the brain biochemistry of patients with SZ in terms of dysregulation of the neurotransmitter network. There is evidence that elevated dopamine concentrations are associated with positive symptoms (i.e., hallucinations, delirium) of the disease. For example, L-DOPA decarboxylase (DDC) is an enzyme involved directly in dopamine and serotonin synthesis and indirectly in noradrenaline synthesis. Therefore, the DDC gene can be considered a candidate gene for schizophrenia and its activity is a good candidate for functional analysis via epigenetic repression.

**Objectives:**

We aimed to create a cellular model with a deregulated DDC gene.

**Methods:**

We constructed two lentiviral vectors, one expressing the dCas9-KRAB-MeCP2 repressor under the control of a synthetic tetracycline inducible promoter, and the other carrying a cassette expressing two sgRNAs with spacers against the DDC promoter. The SH-SY5Y cell line was sequentially stably transduced with both lentiviral constructs, and cells carrying both constructs were selected by the fluorescence of the GFP and RFP reporter proteins encoded in the lentiviral construct backbones.

**Results:**

Methyl-sensitive real-time PCR followed by high-resolution fusion showed methylation of the DDC promoter. Correspondingly, real-time PCR showed a two-fold decrease in DDC expression in SH-SY5Y during tetracycline-induced expression of the CRISPR repressor.

**Conclusions:**

We developed a cellular model to study the contribution of DDC deregulation to SZ-related molecular mechanisms.

**Disclosure of Interest:**

None Declared

